# Treatment progress of spinal metastatic cancer: a powerful tool for improving the quality of life of the patients

**DOI:** 10.1186/s13018-023-03975-3

**Published:** 2023-08-03

**Authors:** Yuliang Zhao, Fei Liu, Wei Wang

**Affiliations:** Department of Bone and Soft Tissue Tumor Surgery, Cancer Hospital of China Medical University, Cancer Hospital of Dalian University of Technology, Liaoning Cancer Hospital and Institute, No. 44 Xiaoheyan Road, Dadong District, Liaoning 110000 Shenyang, China

**Keywords:** Spinal metastasis, Surgical treatment, Minimally invasive treatment, Radiotherapy

## Abstract

Spinal metastasis is a common secondary malignant tumor of the bone, often resulting in spinal cord and nerve root compression, leading to obvious pain and related compression symptoms. This condition has a high incidence and mortality rate. The treatment approach for most patients with spinal metastasis is primarily palliative. Consultation with a multidisciplinary team is widely accepted as a comprehensive treatment approach for patients with spinal metastases. With advancements in research and technology, the evaluation and treatment of spinal metastatic cancer are continuously evolving. This study provides an overview of surgical treatment, minimally invasive treatment, and radiotherapy for spinal metastatic cancer and also analyzes the clinical effects, advantages, and current limitations associated with various treatment approaches.

## Introduction

A tumor is an abnormal cell mass in the body that results from excessive cell division or the failure of the cells to undergo programmed cell death. Tumors can be classified as either benign or malignant. Malignant tumors are characterized by uncontrolled cell growth and have the potential to spread locally or to distant sites. The malignant tumor cells are aggressive and can invade neighboring sites. This spread to distant regions through the bloodstream or lymphatic system is known as metastasis. Metastasis can occur in various parts of the body, with common sites including the liver, lungs, brain, and bones [1].

The spine is a common site for metastasis in malignant tumors, ranking second only to the lung and liver. Approximately 30% of patients with cancer develop spinal metastasis [2]. Various types of cancer can metastasize to the spine, including breast cancer (21%), lung cancer (14%), prostate cancer (8%), and kidney cancer (5%) [3]. Around 12% to 16% of patients experience spinal cord compression symptoms, such as pain, as their initial clinical manifestation [4]. The thoracic spine is the most frequently affected region, accounting for 70% of spinal metastases, followed by the lumbar (20%) and cervical (10%) spines [5]. The rapid growth of metastatic lesions leads to severe bone destruction, which can compress the spinal cord and nerve roots, resulting in pain, pathological fractures, sensory and motor impairments, paraplegia, and other related manifestations [6, 7].

The diagnosis of spinal metastasis is crucial. Early detection and prompt intervention can significantly improve the prognosis and enhance the quality of life. The diagnosis of spinal metastatic cancer encompasses several aspects: (1) *Clinical manifestations* Typically, low back pain serves as the primary clinical manifestation, with pain being the initial presenting symptom in approximately 12–16% of patients. As the tumor grows and affects the vertebral body or compresses the spinal cord and nerve roots, it can cause pain, pathological fractures, impaired bowel movements, sensory and motor dysfunction in the lower limbs, and even paraplegia. Patients often exhibit poor overall conditions and may present with cachexia symptoms such as weight loss, anemia, low fever, and fatigue [6, 7]. (2) *Imaging examination* X-ray, computed tomography (CT), magnetic resonance imaging (MRI), and other imaging modalities are used to determine the tumor location, the extent of invasion and damage, and its relationship with important tissues such as the surrounding spinal cord and nerve roots. (3) *Pathological diagnosis* Puncture biopsy or open biopsy pathology serves as the gold standard for diagnosing spinal metastatic cancer [8].

The diagnosis and treatment of patients with spinal metastatic cancer require a multidisciplinary approach, involving collaboration among various disciplines such as tumor surgery, oncology medicine, tumor radiotherapy, radiology, and pathology departments [9]. This multidisciplinary team (MDT) diagnosis and treatment model allows for comprehensive evaluation and determination of the optimal treatment plan based on the status of the patient, treatment approach, and prognosis. Scoring systems are commonly employed in this process, with the improved Tokuhashi and conventional Tomita scoring systems being widely used. These scoring systems assess various factors to guide treatment decisions. Additionally, the recently proposed neurology, oncology, mechanics, and systematics (NOMS) framework and spinal instability neoplastic scores (SINS) also provide valuable guidance for the treatment [3, 4, 10].

### Tomita score for spinal metastases [11]

**Table:** 

Prognostic factors		Points
Primary tumor	Slow growth (including breast and thyroid)	1
	Moderate growth (including kidneys and uterus)	2
	Rapid growth (including lung and stomach)	4
Visceral metastases	Treatable	2
	Untreatable	4
Bone metastases	Solitary or isolated	1
	Multiple	2

Patients with a Tomita score of 2–3 generally have a longer life expectancy, and surgical treatment aims at achieving long-term local spinal metastasis control. In cases where the Tomita score is 4–5, extensive or marginal tumor resection is performed on the affected vertebral body to achieve mid-term local tumor control. Feasible options include performing marginal or intracapsular tumor resection. However, for patients with a Tomita score of 6–7, short-term palliative treatment is typically recommended, and feasible interventions may include palliative decompression and stabilization surgery. Patients with a score of 8–10 are usually in the end-of-life stage, and drainage treatment may be considered, while surgery is generally not recommended in these patients.

### Tokuhashi score original [12]

**Table:** 

Prognostic factors		Score (points)
General condition (KPS)	Poor (KPS 10–40%)	0
	Moderate (KPS 50–70%)	1
	Good (KPS 80–100%)	2
Number of extraspinal bone metastases foci	≥ 3	0
	1–2	1
	0	2
Number of metastases in the vertebral body	≥ 3	0
	2	1
	1	2
Metastases to the major internal organs	Unresectable	0
	Resectable	1
	No metastases	2
Primary sites of the cancer	Lung and stomach	0
	Kidney, liver, uterus, others, or unidentified	1
	Thyroid, prostate, breast, and rectum	2
	Kidney and uterus	3
	Rectum	4
	Thyroid, mammary, and prostate glands	5
Spinal cord palsy	Complete (Frankel A, B)	0
	Incomplete (Frankel C, D)	1
	None (Frankel E)	2

*KPS*—Karnofsky Performance Status.

In the Tokuhashi revised scoring system, the total score ranges from 0 to 8, 9 to 11, and 12 to 15 points, indicating expected survival periods of < 6, 6–12, and > 12 months, respectively.

The treatment approach for patients with spinal metastatic cancer is primarily palliative and focuses on four main objectives: (1) decompressing the spinal cord and nerve roots to maintain neural function; (2) providing mechanical stability; (3) achieving better local tumor control; and (4) improving the overall condition of the patient [10]. However, it is important to note that surgery has not been shown to significantly improve the survival rates of patients. The treatment approaches for spinal metastatic cancer are diverse, and not all approaches are suitable for every patient. Different treatment modalities have their own characteristics. Common treatment approaches include open surgery, minimally invasive treatment, radiotherapy, and systemic treatment.

Open surgery is indeed a commonly used treatment approach for patients with spinal metastatic cancer. Various surgical techniques can be employed, including separation surgery, posterior laminectomy, posterior total vertebrectomy (en bloc resection), vertebral body replacement, and pedicle screw fixation [13]. Additionally, anterior vertebrectomy and combined anterior and posterior approaches for tumor resection [14] have been utilized. These methods can effectively alleviate pain resulting from spinal cord and nerve root compression, improve neurological symptoms, and correct or prevent vertebral deformities [4, 15].

Minimally invasive treatment has emerged as a newer approach in recent years for managing spinal metastatic cancer, including percutaneous pedicle screw fixation, percutaneous vertebroplasty, and balloon kyphoplasty [16]. Furthermore, significant advancements have been made in the treatment of spinal metastases using various techniques, including radiofrequency ablation (RFA), microwave ablation (MWA), cryoablation, laser interstitial thermotherapy (LITT), endoscope, particle implantation, and minimally invasive decompression [5].

**Fig. 1:**
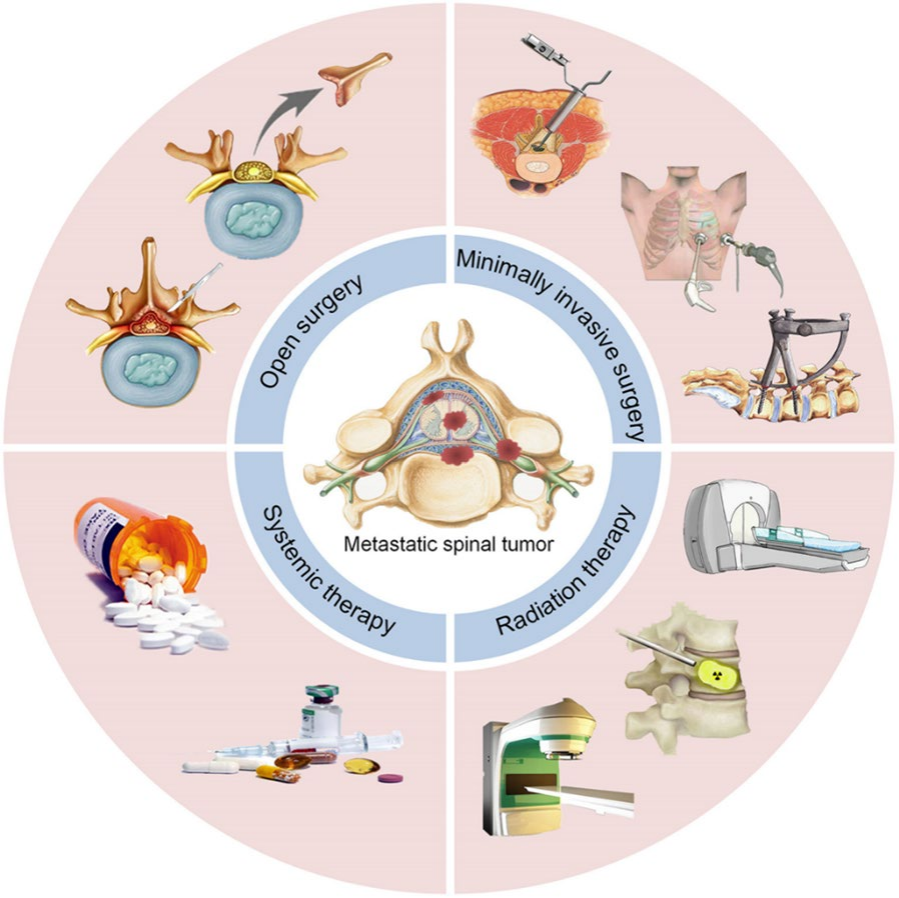
Current status of treatment for spinal metastatic cancer [3]

Radiotherapy plays a pivotal role in the treatment of spinal metastatic cancer, and the emergence of stereotactic radiotherapy has brought significant advancements in tumor control and reduced side effects associated with radiotherapy [9, 17]. This study aims to provide an overview of the metastasis mechanism, decision-making system, open surgery, minimally invasive treatment, and radiotherapy options for spinal metastatic cancer. Furthermore, it will explore the future aspects and development of treatment approaches for spinal metastatic cancer (Fig. 1).

## Treatment decision making

The management of patients with spinal metastatic cancer often involves consultations with MDTs due to the specificity of the disease. Before determining the appropriate treatment approach, it is crucial to evaluate the overall condition of the patient and their life expectancy in order to tailor the treatment plan accurately. Common evaluation systems include the NOMS framework, SINS, improved Tokuhashi, and Tomita scoring systems [3]. Notably, in patients with tumors with a poor prognosis (such as lung cancer), the accuracy of conventional scoring systems like the Tomita score and the improved Tokuhashi score tends to decrease over time [18]. This discrepancy can be attributed to the continuous advancements in treatment approaches for primary tumors. Consequently, these decision-making systems may fail to reflect the improved survival rates and may not directly guide the selection of appropriate treatment approaches for patients with spinal metastasis [4].

In 2010, the Spinal Oncology Research Group launched the SINS, which serves as a consensus-based guide for assessing spinal stability in cases of spinal metastases from tumorous diseases [19]. This scoring system not only establishes unified criteria for evaluating spinal stability but also enhances communication among healthcare professionals during treatment and referrals [20]. Patients with lower SINS scores experience a significant reduction in pain following radiotherapy. Those with higher SINS scores have a higher risk of radiation failure and often require surgery to increase spinal stability [21].

The NOMS Decision-Making Framework was initially proposed in 2006 by [22]. This scoring framework encompasses various treatment approaches such as routine radiation, spinal stereotactic radiotherapy, minimally invasive treatment, and open surgery [23]. The neurological aspect of the framework mainly focuses on assessing the presence of myelopathy or radiculopathy and the extent of spinal cord compression. The epidural spinal cord compression (ESCC) score proves valuable in evaluating this aspect [24]. Oncology-related considerations within the framework aim to predict the response of tumors to existing treatments, with particular emphasis on assessing the radiation sensitivity of tumors [25]. Mechanical evaluation is separate from the decision-making process, which involves evaluating the presence of pathological fractures and determining the appropriate treatment approach. This can be evaluated using the SINS score [26]. Systematics is primarily used to assess the tolerance of patients to the recommended treatment approach [27]. The NOMS and SINS decision-making systems can better adapt to the evolving treatment models compared to the conventional Tomita scoring system and the improved Tokuhashi scoring system and provide timely guidance for patients in selecting appropriate treatment approaches in a timely manner [4].

Advancements in cancer biology research and treatment approaches have promoted the development of decision-making systems for spinal metastasis. There are several potential directions for the future development of these systems. Firstly, the utilization of central or multinational databases. Secondly, integration of histologically specific data. Thirdly, the application of computational methods such as artificial intelligence (AI) learning algorithms. Lastly, combining classification-based and principle-based systems [4] (Table 1).

**Table 1:** Comparison of scoring systems

Scoring systems	Conventional tomita and improved tokuhashi scoring systems	Principle-based NOMS framework
Advantages	1. Can effectively predict patient life expectancy	1. Can display the improvement in survival rate
	2. Can guide specific treatment approaches	2. Can continuously integrate rapidly developing treatment models
		3. Be able to select appropriate treatment approaches for patients in a timely manner
Disadvantages	1. With the improvement of tumor treatment approaches, the improvement in survival rate cannot be displayed	1. Unable to reflect the latest progress in oncology
	2. Unable to integrate rapidly developing treatment models	2. Unable to predict patient life expectancy

## Surgery

### Surgical indications

**Fig. 2:**
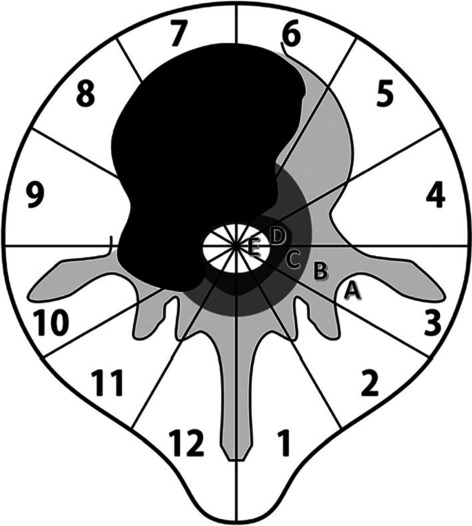
A typical example of isolated spinal metastasis suitable for spinal metastasectomy as per the Weinstein–Boriani–Biagini descriptive system. The alphabets **A**–**E** denote radial levels, or “avers,” of vertebral involvement. **A** Extraosseous paraspinal tissues; **B** intraosseous (superficial); **C** intraosseous (deep); **D** extraosseous (extradural); **E** extraosseous (intradural) [32]

Open surgery remains an important aspect of the treatment of spinal metastatic cancers. Recent clinical research findings suggest that proactive surgical intervention can be used for patients with these tumors [28]. The timing and approach of surgery have a direct impact on the quality of life and survival period of patients [8, 29]. This treatment approach offers several benefits, including: (1) addressing symptoms associated with spinal cord and nerve root compression; (2) enhancing spinal instability; (3) reducing pain; (4) excising epidural tumors before conventional or stereotactic radiotherapy; and (5) facilitating histopathological diagnosis [30]. Open surgery is the preferred treatment for patients experiencing refractory pain, progressive nerve damage, severe pathological fractures, radiotherapy failure, and a high likelihood of cure [31]. Patients undergoing open surgery are typically required to meet certain criteria, including: (1) effective primary tumor control; (2) the absence of disseminated or uncontrollable extraspinal metastatic lesions; and (3) adequate cardiopulmonary reserves to withstand the surgery [32]. As surgery is a palliative treatment, its efficacy depends on the life expectancy of the patient exceeding 3 months, with the potential benefits of improvement outweighing the risks associated with the surgery [2, 30] (Fig. 2).

### Posterior approach laminectomy decompression and total vertebrectomy (en-bloc resection)

Posterior laminectomy is frequently employed for spinal decompression, particularly in patients where there is notable evidence of epidural compression or nerve root compression. This method effectively alleviates pressure on the spinal cord and reduces the risk of nerve root injury [23]. In patients with spinal metastatic cancer localized in the thoracic and lumbar vertebrae, where only one vertebral body is affected and there is good primary tumor control, low malignancy, no significant organ metastasis, and a long life expectancy, total vertebral resection has been proven to achieve good local tumor control. However, it does not guarantee the overall integrity and continuity of the spine. To address this, artificial vertebral bodies or titanium cages are commonly used as substitutes [2, 30, 33]. The most commonly involved regions in spinal metastases are the vertebral body and pedicle, and the removal of these spinal components can impact spinal stability [34]. Consequently, following posterior laminectomy decompression and total vertebral resection, each patient undergoes routine navigation and the placement of pedicle screws. Additionally, preoperative CT scans and bone mineral density scans are used to assess the biomechanical integrity of cancellous bone and determine the need for bone water enhancement. Until 2015, pedicle screws were exclusively made from titanium alloy. Since June 2015, a multi-axial carbon fiber-reinforced carbon or polyetheretherketone pedicle screw system with bone cement reinforcement has emerged [13]. The posterior approach can effectively resect the posterior longitudinal ligament, fibrous ring, and posterior stable structures, thereby controlling bleeding from the epidural venous plexus. However, it does not provide visualization of the ventral structures [35]. In a prospective study conducted by Ibrahim et al. the prognosis of 223 patients with epithelial spinal metastases who underwent surgery, with or without postoperative radiation or chemotherapy, was examined. Postoperatively, the median survival period was 11.7 months, with 71% of patients experiencing improved pain, 53% recovering or maintaining ambulatory activity, and 39% achieving improvement in urinary incontinence. Surgical treatment has effectively enhanced the quality of life by facilitating better pain management, restoring or preserving mobility, and improving sphincter control [36].

### Anterior and combined anterior and posterior approaches for tumor resection

For cervical-thoracic junction and lower lumbar spinal metastases, a combined anterior and posterior approach is typically necessary. In the lower lumbar spine, the proximity of large blood vessels to the vertebral body, as well as the presence of the iliac wings and lumbar plexus, can impede posterior surgical access to the vertebral body. Therefore, in this region, it is necessary to perform a posterior approach before resecting the affected vertebral body through the anterior approach. In cases where tumors extend to the anterior paravertebral region, anterior dissection surgery can assist in the safe execution of posterior surgery. When the lesion is located in the L2–L3 vertebral body, an anterior incision is commonly used to facilitate the separation of the peritoneum from the affected vertebral body [32, 37]. Holman et al. conducted a comparative study on the treatment of lumbar metastatic cancer, comparing posterior decompression, posterolateral fusion, vertebrectomy, and combined anterior and posterior approaches. Their findings indicated that anterior vertebrectomy resulted in less bleeding compared to the posterior approach, and anterior surgery was completely free of infection, whereas the posterior infection rate was 11%. The highest incidence of comprehensive complications was associated with anterior and posterior surgery (75%). Furthermore, combined anterior and posterior surgery showed a higher rate of improvement in neurological function compared to posterior surgery alone (27%, 41%, and 50%, respectively) [15, 38]. However, Terence et al. conducted a new study that demonstrated no significant difference in visual analog scale (VAS) scores, Cobb angle correction, or improvement in neurological function between the combined anterior and posterior approach and the simple posterior approach. Moreover, compared to the simple posterior approach, the combined anterior and posterior approach resulted in a longer total surgical period, higher estimated blood loss, and a longer hospital stay for patients [39] (Table 2).

**Table 2:** Comparison of open surgery approaches

Postoperative comparison	Access selection	
	Combined anterior and posterior approaches	Simple posterior approach
Postoperative VAS score	No significant difference	
Postoperative Cobb angle correction	No significant difference	
Postoperative neurological improvement	No significant difference	
Indications	1. The lesion is located at the cervical thoracic junction and lower lumbar spine and usually requires a combined anterior and posterior approach 2. Suitable for patients with severe damage to the front and middle columns	1. Patients with severe invasion of the posterior column and appendages of the vertebral body 2. The lesion is located in the cervical and thoracic vertebrae at the junction of the upper lumbar spine and non-cervical bear
Surgical period	Long	Short
Intraoperative bleeding	Much	Little
Length of stay	Much	Little
Postoperative complications	More	Less
Fixed segment length	Short	Long
Correction effect of kyphosis deformity	Better	Slightly inferior to the combined anterior and posterior approach
Whether segmental artery ligation is necessary	Yes	No

*VAS*—visual analog scale

### Separation surgery

The separation surgery closely resembles the conventional posterior laminectomy decompression surgery. The specific steps involved in the surgery are as follows: (1) General anesthesia is administered, and the patient is positioned in a prone position. (2) The compression segment of the spinal cord, as well as the upper and lower adjacent vertebral lamina, are exposed. The vertebral lamina in the compression segment is completely removed to alleviate compression. Posterior fixation is then performed with lateral mass screws or pedicle screws in at least two adjacent segments. After posterior decompression, the articular processes are removed through either one or both sides of the pedicle approach to expose the anterior dura mater. To minimize dura mater decompression, the posterior longitudinal ligament is typically removed together (ligament resection is usually conducted at non-tumor segments for better visualization of the separated dura mater). (3) The tumor tissue adhering to the anterior dura mater is meticulously removed. Any tumor-induced frontal vertebral body damage should be scraped off as much as possible, and soft tissues such as intervertebral disks must be removed to achieve complete decompression around the compression dura mater [25].

The purpose of spinal cord separation surgery is to decompress the spinal cord and ensure spinal stability. In cases where the anterior vertebral body of the spinal cord is severely damaged, vertebral resection may be performed, and artificial vertebral bodies or titanium cages can be used to maintain spinal stability. Separation surgery enables the safe separation of the tumor from the spinal cord by a margin of 2–3 mm, which not only alleviates dural compression but also restores the cerebrospinal fluid space surrounding the spinal cord, establishing a radiotherapy gradient area for intraoperative or subsequent radiotherapy [9, 40]. Liu et al. performed separation surgery combined with stereotactic radiotherapy on 52 patients with spinal metastases and found that 46 patients (88.5%) experienced pain relief following the surgery, with an average VAS score of 2.17 points, demonstrating significant improvement compared to preoperative scores (*P* < 0.01). Among them, muscle strength decreased in seven patients, remained unchanged in two, and recovered in 19. Postoperative Frankel neurological function scores and Karnofsky performance scores also showed significant improvement compared to preoperative scores (*P* < 0.01). During the follow-up period of 9–47 months (range, 26.3–18.1), 15 patients died due to deterioration of the primary tumor. Thirteen patients received stereotactic body radiotherapy (SBRT) postoperatively, and 12 of them experienced pain relief. The average VAS score of these 13 patients decreased to 1.64 points, suggesting significant improvement compared to both pre- and postoperative scores (*P* < 0.01), while muscle strength recovered in eight patients. Nelson et al. conducted separation surgery combined with postoperative SBRT on 186 patients and found that this approach had the lowest incidence of postoperative complications when compared to conventional radiotherapy and SBRT [25].

## Minimally invasive treatment

With the advancement of science and technology, various minimally invasive techniques have been continuously applied in the treatment of spinal metastatic cancer. Early minimally invasive techniques are used to treat degenerative spinal diseases, but they have been widely used in the treatment of spinal metastatic cancer. Minimally invasive treatment serves as a favorable option for patients experiencing significant complications, severe malnutrition, severe pain, a weakened immune system, and limited life expectancy. It aids in postoperative recovery, enabling a timely return to primary tumor treatment [41, 42]. Minimally invasive surgery offers several advantages, including reduced soft tissue damage, decreased intraoperative bleeding, and shorter hospital stays [43]. At the same time, in terms of pain relief and improvement of neurological symptoms, the results of minimally invasive surgery are comparable to those of open surgery, and the postoperative infection rate is lower [44]. Common minimally invasive treatments include percutaneous pedicle screw fixation, percutaneous vertebroplasty, percutaneous balloon kyphoplasty, RFA, and MWA. Additionally, minimally invasive decompression, endoscopic technology, LITT, cryoablation, and particle implantation have shown promising therapeutic effects for spinal metastatic cancer [3, 15, 16].

### Percutaneous pedicle screw fixation

Percutaneous pedicle screw fixation serves as an effective alternative to open surgery for patients with pathological fractures causing spinal instability who are unable to undergo open surgery or bone cement filling. By utilizing the expertise of orthopedics, biomechanical stability can be achieved at various spinal levels [45, 46]. However, this approach may lead to certain skin complications and might not fully alleviate the pain resulting from spinal metastatic cancer. Hence, it is primarily used for patients awaiting surgery and those ineligible for surgery [43]. In cases of mechanically unstable fractures caused by radiotherapy-sensitive tumors (like lymphoma, multiple myeloma, breast cancer, and prostate cancer) accompanied by evident epidural compression, this treatment can be combined with radiotherapy to address the problem of epidural tumor components [16]. A comparative study by Yue et al. examined 102 patients with spinal fractures treated with percutaneous and open pedicle screw internal fixations and found that compared to open pedicle screw internal fixation, percutaneous pedicle screw internal fixation yielded a more significant therapeutic effect, decreased intraoperative bleeding, and a shorter hospital stay. This approach significantly improved the condition of the vertebral body, decreased the incidence of postoperative complications, accelerated patient recovery, and improved safety levels [47].

### Percutaneous vertebroplasty and kyphoplasty

Percutaneous vertebroplasty and kyphoplasty are techniques for injecting polymethylmethacrylate (PMMA) into the vertebral body under the guidance of an X-ray or CT. Vertebroplasty entails the direct injection of bone cement into the affected vertebral body, while kyphoplasty involves restoring the compressed vertebral body using an inflatable balloon before the injection of bone cement [48]. These two techniques are suitable for patients experiencing persistent pain with a life expectancy of less than 3–6 months or who are unable to tolerate standard open surgery [49]. The primary goal of these procedures is pain reduction, achieved through mechanisms such as chemical toxicity, thermal necrosis effects, and increased vertebral body stability in cases of pathological fractures. Enhancing the vertebral body’s stability serves as their main mechanism of action [50, 51]. A study by Sun et al. [52] showed that the CT scan results of patients before vertebroplasty and 1 week after the procedure showed a certain extent of recovery in the anterior, middle, and posterior heights of the affected vertebral bodies. Research conducted by Hadjipavlou et al. showed that the postoperative pain relief for vertebroplasty and kyphoplasty ranged from 75.9 to 92.5% and 75.6 to 98.2%, respectively, with no significant difference between them [53]. Cement leakage is the most common complication associated with vertebroplasty, with some studies indicating that up to 75% of patients experience varying degrees of cement leakage following the procedure [54]. Percutaneous kyphoplasty, a variant of vertebroplasty, employs a siphon balloon to restore vertebral height, reduce the risk of bone cement penetration, and address vertebral collapse [45]. However, this method is not suitable for patients with posterior wall involvement and spinal cord compression, as they require additional instruments to maintain spinal stability [55].

### RFA

RFA of tumors has been widely used worldwide. Alongside its common applications in liver, lung, kidney, and thyroid cancers, RFA for bone tumors is also rapidly emerging [56]. Rosenthal et al. first reported the use of RFA in the treatment of osteoid osteomas in bones in 1992 [57]. Nowadays, RFA for the treatment of bone metastases is gradually maturing. RFA is a minimally invasive percutaneous procedure that involves the insertion of electrodes into the vertebral body of a tumor under the guidance of fluoroscopy or CT. By using the heat generated by high-frequency alternating current (typically ranging from 300 to 600 kHz), tumor cells are damaged, leading to heat-induced protein denaturation and subsequent coagulative necrosis [16, 58, 59].

In the past 5–10 years, RFA has been used as an alternative palliative treatment for patients with spinal metastatic cancer, primarily to alleviate pain. Absolute contraindications for RFA are rare, including lack of safe passage, acute immunosuppression, local or systemic infections, uncorrected coagulation dysfunction, and patient refusal to consent. Relative contraindications involve very large lesions and proximity to sensitive structures that cannot be effectively monitored or protected. The most common relative contraindications in the spine are unstable fractures and metastatic epidural spinal cord compression [60]. Specific limitations of RFA include its sensitivity to radiation effects and limited efficacy in osteoblastic lesions. While the presence of a metal surgical fixation device does not pose an absolute prohibition, utmost caution is required when placing the probe near it to avoid potential adverse heating and electrical effects [60]. In the context of concerns regarding adjacent nerve damage, Buy et al. described special nerve thermal protection techniques, including the use of epidural or neural pore thermocouples and the injection of carbon dioxide or cooled 5% glucose water [61]. In a retrospective study conducted in 2014 involving 92 patients with spinal metastatic cancer who underwent RFA, significant pain relief was reported during follow-up visits at 1 week, 1 month, and 6 months postoperatively. Regarding the use of postoperative painkillers, 54% of patients reported a decrease in their usage, 30% reported no change, and 16% reported an increase [62]. Among the 92 treated patients, 34 received detailed surgical information. In a retrospective study of these 34 patients conducted by Praveen et al. the ablation time for each treated lesion ranged from 55 to 653 s, with an average ablation time of 361 s. Each lesion was treated with an average of 4.3 overlapping ablation zones. The average temperature recorded at the proximal thermocouple on the electrode (representing the temperature reading of the outermost portion of the ablation zone) was 50 °C, and the average temperature recorded at the distal thermocouple was 73 °C. Twenty-one out of 34 patients (62%) received treatment for injuries located at the back of the vertebral body [62]. While RFA can provide limited pain relief, it does not improve neurological function or prevent pathological fractures. For stable pathological vertebral compression fractures, RFA is often combined with vertebroplasty to enhance neurological function and reduce pain [63, 64].

## MWA

MWA operates by using electromagnetic fields (at frequencies of 915 MHz or 2.45 GHz) through an antenna directed at the target tumor. The electromagnetic field induces continuous rearrangement of the dipole in its primary direction, forcing the polar molecules (primarily water) within the tissue to continuously rearrange with the oscillating electric field. This process increases the kinetic energy of the molecules, thereby increasing the temperature of the tissue and causing subsequent coagulation necrosis [65]. Tissues with abundant water content, such as solid organs and tumors, are particularly responsive to this heating process [66]. MWA has many advantages over conventional RFA. Microwave energy is capable of generating faster heating on larger tissues, and it is less sensitive to radiator effects. Furthermore, it can effectively heat tissues with high impedance, such as lungs or burned, dry tissues, and is capable of producing very high temperatures, often exceeding 100 °C. The ability to use multiple transmitters is another favorable aspect of MWA, which does not require grounding pads or other auxiliary components [65]. Theoretically, MWAs are relatively insensitive to tissue characteristics such as impedance and perfusion, so MWA energy can penetrate all biological tissues, making it particularly potent in generating large ablation zones within minutes [67]. Consequently, MWA has found widespread application in the treatment of liver tumors that require substantial ablation regions to optimize local tumor control [66].

Due to the relative dielectric constant of bones, microwaves may be less influenced by tissue heating and drying, allowing for deeper penetration and more effective heating. However, the use of MWA in the treatment of spinal metastases is very limited. Chen et al. conducted MWA under the guidance of CT on 91 patients with 140 metastatic vertebrae, achieving technical success in all patients. One month after treatment, compared to before treatment, the median VAS score decreased by an average of 3, and the average morphine dose decreased by 56.70 mg. The Oswestry disability index score also showed improvement (*P* < 0.01) [68]. Treating spinal metastatic cancer with MWA carries a greater risk of injury to susceptible nerve elements such as the spinal cord and nerve roots due to their proximity. Injuries to the spinal cord and nerve roots can lead to relatively serious complications. Common protective measures include real-time monitoring of peripheral nerve temperature, perineural and epidural injections of carbon dioxide or 5% glucose aqueous solutions, and the use of low-power and repetitive short ablation cycles (30–90 s) to control the diffusion of hot spots [69, 70].

Chen et al. performed intermittent 30 W MWA for 4.5 min and injected bone cement into the metastatic vertebral body. Postoperative imaging showed residual tumors in the epidural space causing compression on the spinal cord without any cement leakage into the spinal canal. Postoperatively, the patient received mannitol (125 mL, intravenous injection, 8 tablets/h, lasting for 3 days), glucocorticoids (methylprednisolone, 200 mg/day, intravenous injection, lasting for 3 days, with a subsequent 3% every 20 days), and radiotherapy (30 Gy, 10 times/min). Three patients with epidural compression (one out of 49; 2.0%) experienced a grade 1 nerve injury. They developed partial hemiplegia (3/5 motor intensity) postoperatively, but their nervous system function returned to normal 1 month after radiotherapy [68]. Regarding MWA power and time, all included studies exhibit significant differences, highlighting the lack of novelty of this procedure, where the ablation protocol has not yet been standardized. Khan et al. used very low power (an average of 13 W), with an average ablation time of 286 s for spinal tumors, corresponding to approximately 3.9 kJ of delivered energy [70]. Conversely, Kastler et al. [69] applied high power (an average of 60 W) in the same clinical setting, with an average ablation time of 264 s, equivalent to approximately 15.8 kJ.

### Other minimally invasive techniques

Cryoablation, similar to RFA, involves the insertion of a cryoprobe into the affected vertebral body under image guidance through the skin, using extremely low temperatures to eliminate cells [71]. Cryoablation is primarily used for spinal metastatic cancers with substantial soft tissue components. When dealing with osteogenic lesions, cryoablation demonstrates superior efficacy compared to RFA, as the presence of thickened bone can impede the efficacy of the high-frequency alternating current used in RFA [58].

LITT is a new treatment approach for patients with spinal metastatic cancer [72]. Serving as an alternative to open surgery, it delivers thermal energy to eliminate tumor cells while being guided by real-time magnetic resonance imaging monitoring [73]. Tatsui et al. (2015) reported the initial utilization of LITT in treating spinal metastatic cancer. Their research showed a reduction in the average thickness of epidural tumors, a significant alleviation of epidural spinal cord compression, decreased pain in the patients, and an improvement in their overall quality of life when compared to conditions before the treatment [74].

With the emergence of stereotactic radiotherapy, its high tumor control rate and low incidence of complications have reduced the need for extensive tumor resection [75]. Minimally invasive decompression, as an alternative to open surgery, offers reliable symptom relief for patients while minimizing soft tissue damage, thereby promoting patient recovery and reducing the occurrence of complications [76].

Endoscopic technology and Da Vinci robots have been widely used in cardiothoracic surgery due to their ability to minimize chest damage and improve visualization [20]. They are particularly useful in cases requiring a combined anterior and posterior approach [5]. However, due to the high technical requirements of this method, its widespread use is still limited.

Particle implantation under the guidance of CT is another commonly used treatment approach for spinal metastatic cancer. By directly implanting a radiation source into the tumor site, it enables the precise delivery of high radiation doses while preserving surrounding healthy tissue. It is commonly used in patients who cannot tolerate other treatments or as an adjuvant treatment in combination with other treatments [77].

## Radiotherapy

**Fig. 3:**
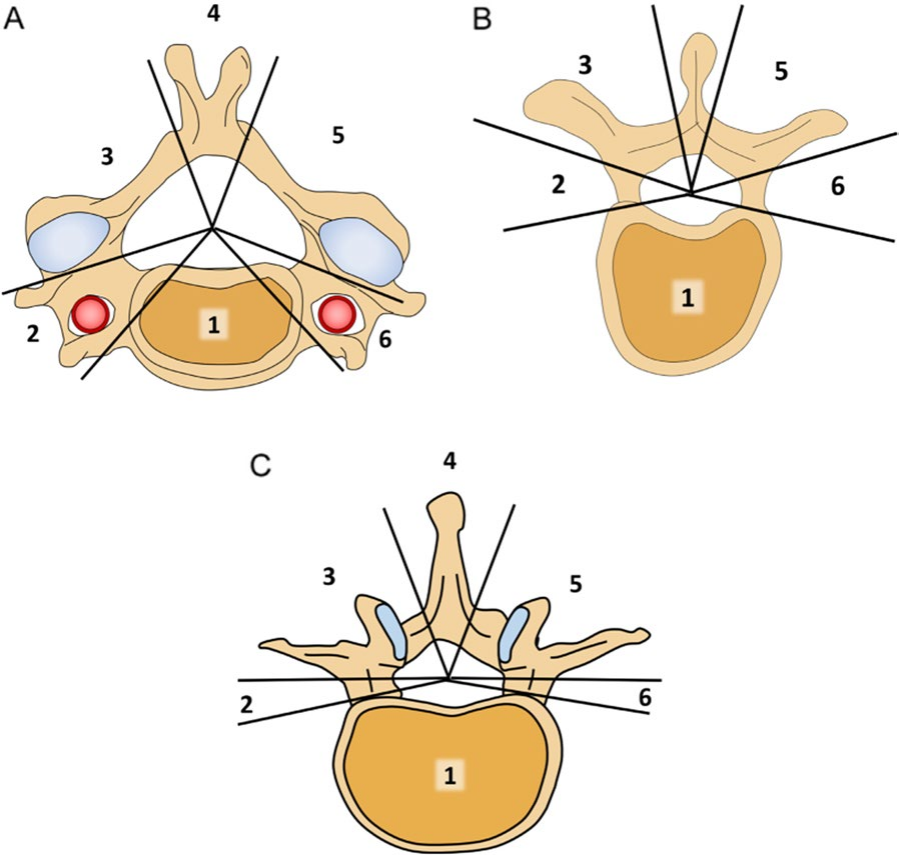
International spine radiosurgery consortium anatomic classification system for consensus target volumes for spine radiosurgery y[83]

Radiotherapy is considered one of the palliative treatments for patients with spinal metastatic cancer [78]. Over the past few decades, significant advancements have been made in radiotherapy for spinal metastatic cancer [79], leading to significant effects in pain relief, local tumor control, and neurological function recovery [80]. Radiotherapy is a non-invasive treatment that results in minimal and temporary side effects, making it well tolerated by patients [81]. The indications for radiotherapy in spinal metastatic cancer include the following: (1) patients who are not suitable candidates for surgery; (2) multiple vertebral segments or extensive vertebral appendage involvement; and (3) tumors that are sensitive to radiotherapy [82]. Radiotherapy can be performed through conventional external beam radiotherapy (EBRT) or SBRT, depending on the treatment goals or unique, specific factors of the patient [2] (Fig. 3).

### Conventional EBRT

Conventional EBRT is the most widely used form of radiotherapy and can be administered as a standalone treatment or in combination with other treatments [9]. EBRT is a two-dimensional technique primarily targeting the affected vertebral bodies and adjacent upper and lower vertebral bodies [46]. However, the broad range of irradiation in EBRT increases the risk of unnecessary irradiation of adjacent normal tissues. Consequently, the irradiation dose of this method is limited to minimize toxicity to surrounding tissues, often necessitating multiple treatment sessions [84, 85]. The efficacy of EBRT largely relies on the radiation sensitivity of tumor subtypes [86]. Tumors that generally respond well to EBRT include various hematological tumors (such as lymphoma, multiple myeloma, and plasmacytoma) as well as certain solid tumors (including breast cancer, prostate cancer, ovarian cancer, and seminoma) [20, 87]. However, most solid tumors exhibit poor responses to EBRT, such as renal cell carcinoma, non-small cell lung cancer, thyroid cancer, hepatocellular carcinoma, melanoma, and sarcoma [85, 87].

### SBRT

Compared to conventional radiotherapy, SBRT is a more novel and targeted radiotherapy approach [88]. SBRT has specific requirements, including a small and well-defined target, high conformability of the radiation dose, and an accurate dose delivery system [9]. The emergence of SBRT has revolutionized the treatment approach for spinal metastatic cancer, impacting surgical indications and the types and scope of surgery. SBRT overcomes the resistance of tumors to radiotherapy by safely delivering high doses of radiation to tumors while minimizing the amount of radiation to surrounding organs [89]. Unlike the known mechanism of cell death induction by conventional EBRT, the efficacy of SBRT in overcoming the resistance of tumors lies in providing additional tumor-killing pathways through high-dose radiation [90, 91]. Studies have shown that high-dose radiation can effectively eliminate tumor cells and disrupt newly formed tumor blood vessels, as these vessels are particularly sensitive to ionizing radiation [92]. Another mechanism of cancer eradication by SBRT involves stimulating tumor antigen cells to generate specific immune responses and induce cell apoptosis [93].

Yurday et al. [17] analyzed 78 patients with spinal metastases who underwent SBRT and found that their local control rate was 88% and the vertebral compression fracture rate was 4%. Sprave et al. conducted a non-blind randomized trial to compare the difference in pain relief between stereotactic radiosurgery (SRS) with a dose of 24 Gy and conventionally fractionated radiotherapy (CRT) consisting of 10 fractions of a total dose of 30 Gy for painful spinal metastases. The results showed no significant difference in VAS scores between the two groups at 3 months (*P* = 0.13). However, during this period, the VAS score in the SRS group decreased at a faster rate (*P* = 0.01). At 6 months, the VAS score in the SRS group was significantly lower than that in the CRT group (*P* = 0.002) [94]. The advantages of SBRT include: (1) avoiding excessive radiation to non-tumor regions; (2) having a short treatment period that minimally interferes with other treatments; (3) effectively treating tumors that are not sensitive to conventional radiotherapy; (4) providing long-term pain relief; and (5) being a non-invasive treatment approach [9]. Furthermore, SBRT has the potential to preserve more bone marrow, which is important for chemotherapy tolerance [95].

However, it should be noted that radiotherapy has its limitations and associated complications. For example, if the pain is due to spinal instability, radiotherapy alone may not provide sufficient pain relief [43]. Additionally, radiotherapy is associated with the risk of vertebral compression fractures, and factors such as extreme compression fractures, osteolytic tumors, and dislocations increase the risk of spinal compression fractures caused by SRBT [96]. Furthermore, there is currently no standardized radiotherapy regimen for patients with spinal metastatic cancer. Different radiotherapy schedules and dose regimens are being used worldwide, and there is a lack of comparative studies demonstrating the superiority of one approach over another [82].

## Summary and prospect

The spine is a common metastatic site for malignant tumors, with approximately 30% of patients with cancer experiencing spinal metastasis. Due to the specificity of the spinal cord’s location adjacent to the nerve roots, the clinical manifestations of patients with spinal metastatic cancer are often evident. Tumors can cause spinal cord and nerve root compression by destroying normal spinal structures, leading to pain, dysfunction, sensory impairment, and, in severe cases, loss of bowel function and paraplegia. As treatment technology and approaches have advanced, evaluation systems for spinal metastatic cancer have also evolved. The accuracy of the conventional Tomita and improved Tokuhashi scoring systems is diminishing over time. The SINS score and NOMS framework are more widely used in the evaluation of spinal metastatic cancer. In future, new decision-making systems based on big data and using computer AI may emerge, enabling better assessment of treatment approaches and patient prognosis. The role of consultations with MDT in the diagnosis and treatment of spinal metastatic cancer is constantly strengthening. While the treatment of spinal metastatic cancer is primarily palliative, open surgery remains the preferred approach as it can more effectively alleviate the compression of the lesion and relieve associated symptoms. The ongoing development of minimally invasive treatments has provided additional options for patients with spinal metastatic cancer. In future, surgical approaches for spinal metastatic cancer will be more prone to low injury and high benefit, and emerging technologies such as laser interstitial thermotherapy and Da Vinci robots will be widely used. Although conventional radiotherapy is still commonly used, SRBT is gradually gaining acceptance among treatment teams due to its high safety and efficacy.

## Data Availability

None.
